# The role of endolysosomal progranulin and TMEM106B in neurodegenerative diseases

**DOI:** 10.1186/s13024-025-00873-6

**Published:** 2025-07-26

**Authors:** Hideyuki Takahashi, Stephen M. Strittmatter

**Affiliations:** Cellular Neuroscience, Neurodegeneration, Repair, Departments of Neurology and of Neuroscience, Yale University School of Medicine, New Haven, CT 06536 USA

**Keywords:** Progranulin, TMEM106B, Frontotemporal Lobar degeneration, Alzheimer’s disease, Parkinson’s disease, Aging, Endolysosome, Glycosphingolipid, Amyloid fibrils, GBA1

## Abstract

Although different neurodegenerative diseases are defined by distinct pathological proteins, they share many common features including protein aggregation. Despite this commonality, most current therapeutic approaches in the field, such as anti-aggregate antibodies, are focused on individual diseases or single neuropathologies with only limited success. The endolysosomal proteins progranulin and TMEM106B were both initially associated with frontotemporal lobar degeneration but have subsequently also been linked to other neurodegenerative diseases. Thus, these proteins are predicted to participate in common pathogenic pathways shared across various neurodegenerative diseases. Importantly, recent discoveries of TMEM106B amyloid fibrils in varied neurodegenerative diseases and glycosphingolipid regulation by progranulin and TMEM106B further support their central roles in cross-disease neurodegenerative mechanisms. This review summarizes recent advances in progranulin and TMEM106B function within the endolysosomal system and neurodegenerative diseases. It describes preclinical models and therapeutic approaches for progranulin- and TMEM106B-associated diseases. We also discuss future direction leading to novel alternative therapies targeting shared mechanisms in neurodegenerative diseases.

## Background

Neurodegenerative diseases such as Alzheimer’s disease (AD), Parkinson’s disease (PD), frontotemporal lobar degeneration (FTLD), and amyotrophic lateral sclerosis (ALS), share many pathological features, including neuronal and synaptic loss, neuroinflammation, and accumulation of protein aggregates in the brain [1]. In addition, recent studies using human postmortem brains have shown that in addition to a primary proteinopathy, concomitant proteinopathies are frequently observed in patients with neurodegenerative diseases and influence their clinical course [2–4]. Despite these facts, current research and therapeutic approaches in the field have been focused on single protein neuropathologies, such as Aβ, tau, and -synuclein, with only limited success.

The endolysosomal system is essential for maintaining cellular proteostasis and its dysfunction is thought to be involved in accumulation of pathological proteins in various neurodegenerative diseases. Human genetic studies have identified numerous genes in the endolysosomal system associated with AD, PD, FTLD, and ALS, highlighting an importance of the endolysosomal system in a wide range of neurodegenerative diseases [5, 6]. The endolysosomal proteins progranulin (PGRN) and TMEM106B were initially implicated in FTLD with TAR DNA-binding protein 43 (TDP-43) inclusions (FTLD-TDP) [7–9]. Importantly, *TMEM106B* variants were also reported to contribute to genetic risk for FTLD-TDP in individuals with *GRN* mutations [7], suggesting a potential functional interaction between PGRN and TMEM106B in the endolysosomal system. Subsequently, these two proteins were also linked to many other neurodegenerative diseases including AD and PD [10, 11]. Thus, PGRN and TMEM106B may play a central role in common pathological mechanisms shared across various neurodegenerative diseases, enhancing their potential impact as therapeutic targets with broad application. In this review, we provide an overview of PGRN and TMEM106B in neurodegenerative diseases and the endolysosomal system. We describe recent discoveries of amyloid fibrils of C-terminal fragments of TMEM106B and lipid regulation by PGRN and TMEM106B. Advances in preclinical disease models as well as therapeutic targeting of PGRN- and TMEM106B-associated neurodegenerative diseases are reviewed. Finally, we consider future directions for PGRN and TMEM106B research designed to develop novel therapies targeting common mechanisms of multiple neurodegenerative diseases.

## PGRN-associated neurodegenerative diseases

Heterozygous mutations in the *GRN* gene that result in PGRN haploinsufficiency cause FTLD [8, 9], the second most common cause of dementia in people under the age of 65 [12]. FTLD affects the frontal and temporal lobes of the brain, resulting in progressive changes in behavior, personality, and/or language [12, 13]. The two major pathological subtypes of FTLD are FTLD-tau characterized by tau pathology and FTLD-TDP associated with TDP-43 pathology, although 5–10% of patients may have inclusions immunoreactive for FUS, EWS, and TAF15 (FTLD-FET) [13]. *GRN* mutations account for approximately 5–25% of familial FTLD and are the second most common cause of inherited FTLD-TDP, after a CCCCGG hexanucleotide expansion in the non-coding region of *C9orf72* [13]. The penetrance of *GRN* mutations is incomplete and reportedly age dependent with only 50% of carriers being affected by age 60 and 90% by age 70 [14]. Neuropathological analysis has shown that *GRN* mutations are exclusively associated with FTLD-TDP type A pathology, which is characterized by abundant cytoplasmic inclusions and short thick dystrophic neurites in the superficial cortical layers [12]. A significant increase in microgliosis has also been reported in the brain of FTLD patients with *GRN* mutations (FTLD-*GRN*) [15, 16]. In addition, recent single-nucleus RNA sequencing studies have revealed neurovascular dysfunction and astrocytic pathology in the brain of FTLD-*GRN* [17, 18]. At least 140 different loss-of-function mutations have been found in FTLD patients, and most of them lead to premature termination codons (PTCs) either directly due to nonsense mutations or indirectly through frameshift by splice-site mutations or small deletions and insertions. The net result is destruction of mutant *GRN* mRNA by nonsense-mediated mRNA decay with a consequent 50% loss of functional PGRN [19]. In addition to mutations leading to PTCs, a number of missense mutations have been also reported. Although for most of the missense cases, their contribution to the pathogenesis is not well defined, the W7R and A9D mutations in the signal sequence have been shown to affect mRNA stability, sorting, and/or secretion of PGRN [20, 21].

**Fig. 1:**
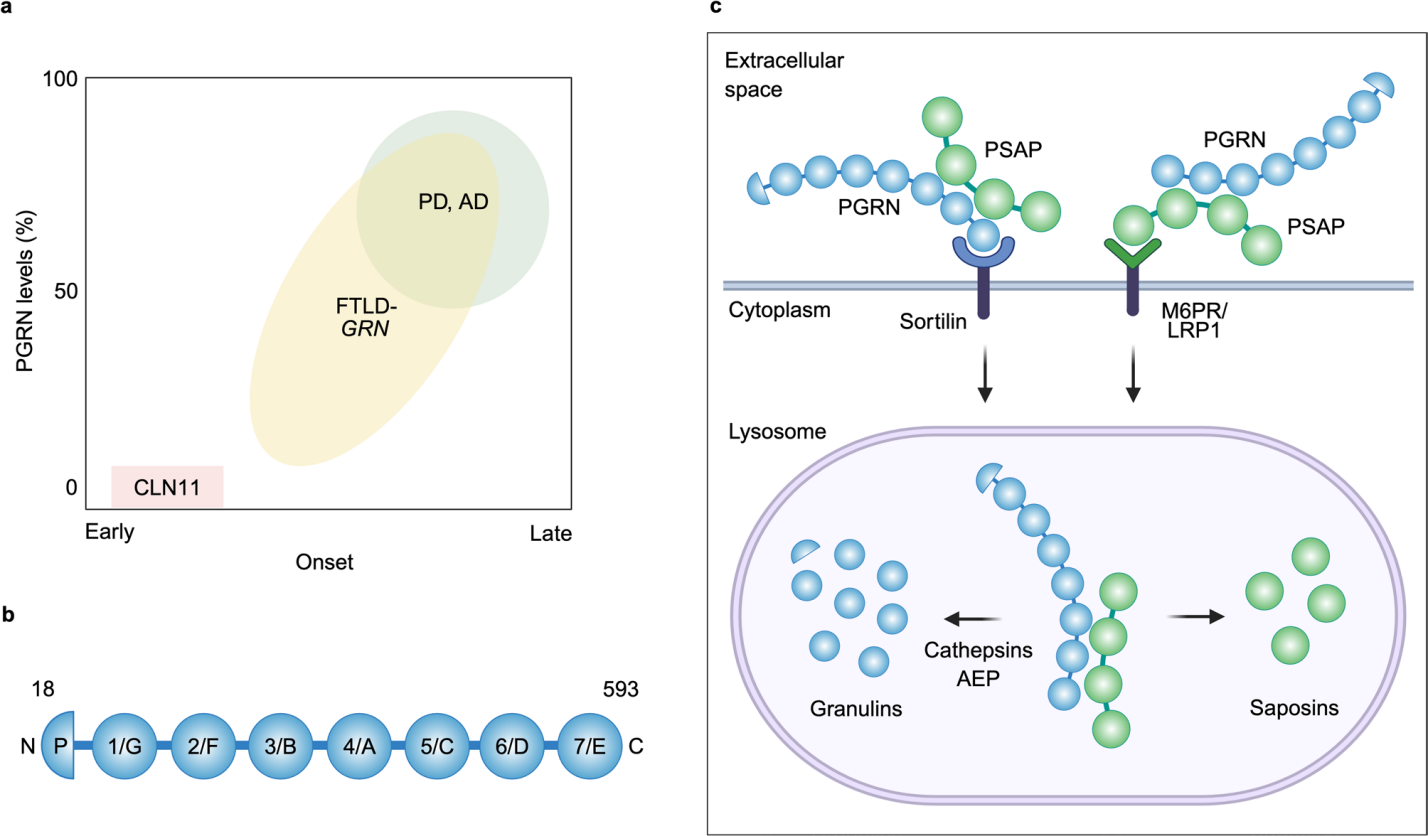
Schematic illustration of structure and cellular uptake of PGRN. **a**, PGRN-associated neurodegenerative diseases. Haploinsufficiency (~50% loss) of PGRN causes FTLD, the second most common cause of dementia in people under the age of 65, while complete loss of PGRN causes the lysosomal storage disorder CLN11 with the age of onset of ~15 years, although rare homozygous GRN mutations were reported to cause FTLD with the age of onset of ~50 years. In addition, several GRN variants increase risk for AD and PD, which usually develop after age 65 and 60, respectively. A GRN variant is reported to cause 10-20% reduction in PGRN levels. Furthermore, heterozygous GRN mutations have been found in a substantial number of AD and PD patients. **b**, Schematic representation of PGRN. Human PGRN is a highly glycosylated protein composed of 7.5 cysteine-rich granulin domains (A to G and P) that are connected by short linker regions. P represents paragranulin. Amino acids 1 to 17 are the signal sequence. Granulins numbered 1 through 7 are based on the UniProtKB database: P28799. **c**, Schematic illustration of lysosomal delivery of PGRN and PSAP via their receptors. Extracellular PGRN binds to cell surface sortilin receptor through its C-terminal tail and is delivered to lysosomes, where PGRN is processed into granulins by cathepsins and AEP. PGRN also binds to PSAP extracellularly through the linker region between saposins B and C and can be delivered to lysosomes via the PSAP receptors M6PR and LRP1. The PGRN-PSAP complex may also be important to facilitate lysosomal delivery of extracellular PSAP via sortilin. In the lysosome, PSAP is processed into saposins, which are involved in glycosphingolipid degradation. Figure was created with BioRender.com.

While PGRN haploinsufficiency causes FTLD-TDP, complete loss of function of PGRN due to homozygous *GRN* mutations leads to neuronal ceroid lipofuscinosis (CLN11), a rare lysosomal storage disorder characterized by excessive accumulation of lipofuscins in the lysosome [22]. Clinical features of CLN11 include cerebellar ataxia, epilepsy, visual loss, and progressive cognitive decline with the age of onset of ~ 15 years [22–27]. Importantly, a recent study however has reported six patients carrying homozygous *GRN* mutations with divergent phenotypes and variable ages of onset, including some presenting classical CLN11 symptoms at early age of onset and others showing a distinct delayed phenotype of frontotemporal dementia after 40 or 50 years, suggesting that CLN11 and FTLD-TDP are extreme phenotypes of a common spectrum of disorders caused by biallelic *GRN* mutations [28] (Fig. 1a).

Previous genetic studies including genome-wide association studies (GWAS) have suggested that common *GRN* variants are associated with increased risk for AD and PD [29–34]. In addition, *GRN* mutations have been found in a substantial number of AD and PD patients [14, 35–46] (Fig. 1a). A recent study has also shown an association of *GRN* mutations with Lewy body dementia (LBD) [47]. AD is pathologically characterized by accumulation of extracellular amyloid plaques composed of amyloid-β (Aβ) peptides and intracellular neurofibrillary tangles formed from hyperphosphorylated tau protein in the brain [48]. PGRN is known to be up-regulated in dystrophic neurites and microglia near amyloid plaques, while no or very weak PGRN immunoreactivity has been detected in neurofibrillary tangles in AD [49–51]. However, in human subjects of the Alzheimer’s Disease Neuroimaging Initiative (ADNI), the *GRN* rs5848 AD risk variant, which causes at least 10–20% reduction of PGRN protein levels, has no significant effects on florbetapir PET amyloid imaging or cerebrospinal fluid (CSF) Aβ levels, whereas it is associated with increased CSF tau levels [52], suggesting a potential effect of PGRN deficiency on tau pathology, rather than amyloid pathology. Importantly, accumulation of tau and α-synuclein, in addition to TDP-43 inclusions, is also found in the brain of several *GRN* mutation carriers [39, 45, 53–56]. In addition to AD and PD/LBD, *GRN* variation has also been implicated in Gaucher disease [57], FTLD-TDP and/or motor neuron disease with *C9orf72* repeat expansions [58], ALS [59], and limbic-predominant age-related TDP-43 encephalopathy neuropathological change (LATE-NC) [60].

## TMEM106B-associated neurological diseases

**Fig. 2:**
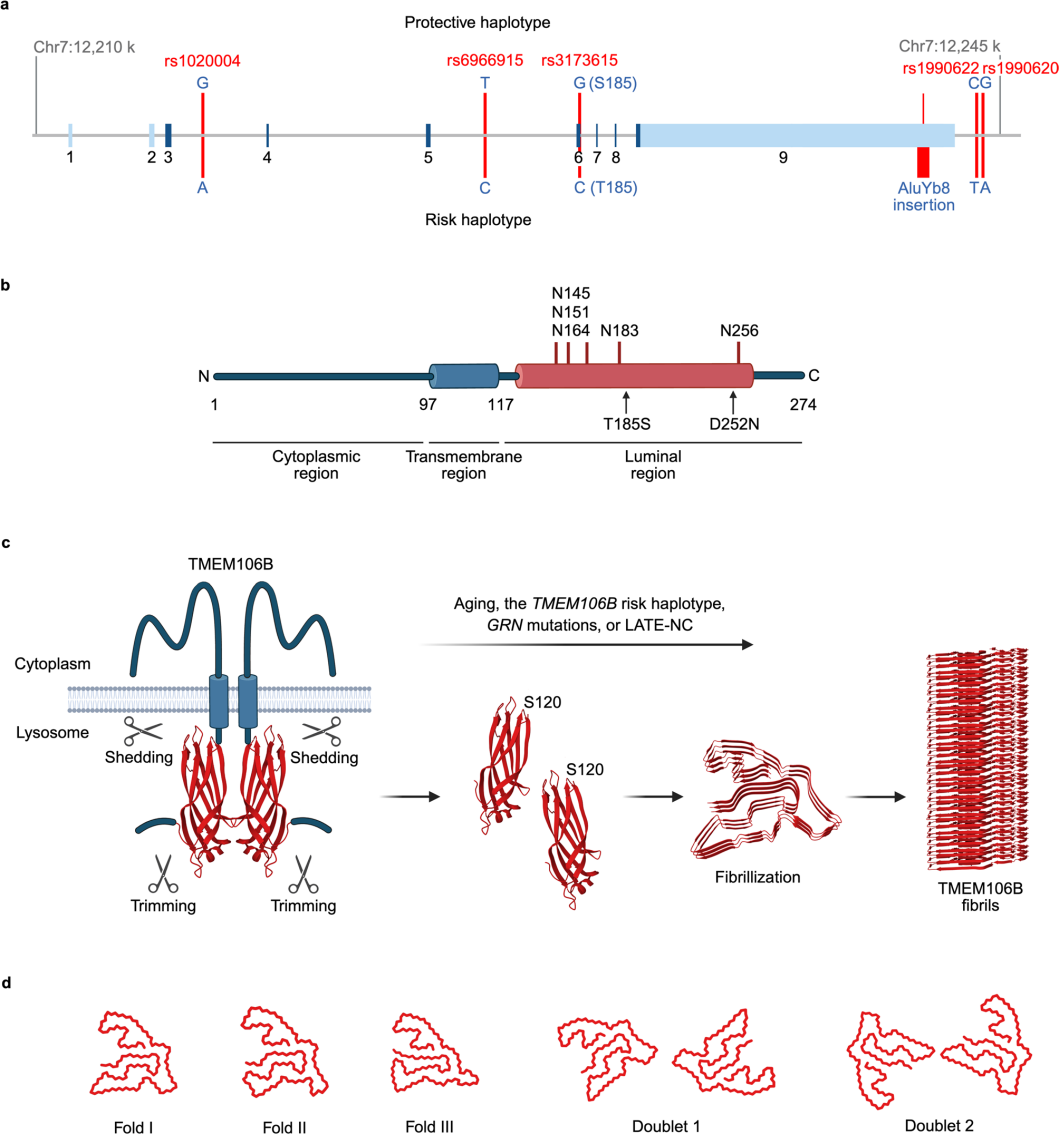
Schematic illustration of structure and amyloid formation of TMEM106B. **a**, TMEM106B genomic region on chromosome 7p21. Exons (1-9) are indicated by blue boxes and the coding regions are labeled in dark blue. The major SNPs associated with neurodegenerative diseases are shown with protective/minor and risk/major alleles. Multiple SNPs near and in the TMEM106B gene, including rs1990620, rs1990622, rs3173615, rs6966915, and rs1020004, are in strong LD, constituting two common TMEM106B haplotypes, one associated with increased disease risk, and the other with a protective effect. AluYb8 insertion is found in 3’ UTR of the TMEM106B risk haplotype. **b**, Schematic representation of TMEM106B. The 274-amino acid human TMEM106B consists of an N-terminal intrinsically disordered cytoplasmic region, a single-pass transmembrane region, and a C-terminal luminal region with five glycosylation sites (N145, N151, N164, N183, and N256). T185S and D252N amino acid substitutions associated with FTLD and HLD are indicated. **c**, Amyloid fibril formation of CTF of TMEM106B. TMEM106B forms homodimers and undergoes shedding and C-terminal trimming by unknown lysosomal enzyme(s). It is currently unknown whether dimerization affects the processing of TMEM106B and what protease(s) mediate the processing to form the fibrils. A precise cleavage between resides 119 and 120 appears to be required for the amyloid fibril formation because Ser120 is buried in the fibril core, leaving no space for the other N-terminal residues. Upon fibrillization, the luminal region of TMEM106B undergoes a conformational change from a structure with a ubiquitous 7-bladed β sandwich fold (PDB: 8B7D) to ones with a five-layered fold consisting of 17-19 β-strands (PDB: 7QVC). The fibril formation is age-dependent and may be promoted by the TMEM106B risk haplotype, GRN mutation, or under LATE-NC condition. Note that, for the sake of simplicity, glycosylation is omitted from the illustration but the glycosylation sites within the fibril core (N145, N151, N164, and N183) are reported to be fully glycosylated. **d**, In TMEM106B fibrils, three major filament folds (I-III) and two doublet polymorphisms (1,2) have been reported. Unlike other amyloid proteins, no clear relationships between the filament folds and diseases were found. Folds I-III and doublets 1-2 are extracted from PDB: 7QVC, 7QWG, 7QWM, 7QVF, and 7SAS, respectively.Figure was created with BioRender.com.

The *TMEM106B* gene was initially identified in 2010 by a GWAS as a genetic risk factor for FTLD-TDP [7]. For the top single-nucleotide polymorphism (SNP) rs1990622 (*P* = 1.08 × 10^–11^) (Fig. 2a), the minor C-allele conferred protection with an odds ratio of 0.61 (minor allele frequency 32.1% in cases and 43.6% in controls). Importantly, this study also reported that the disease-modulating effect was especially pronounced in FTLD patients with *GRN* mutations [7]. Subsequent studies have confirmed that *TMEM106B* is a strong modifier for FTLD-TDP caused by *GRN* mutations [61–66]. Remarkably, it has been consistently reported that homozygotes for *TMEM106B* protective minor alleles are rarely found in symptomatic FTLD patients with *GRN* mutations [61, 62, 64–66]. These results suggest that homozygosity for the *TMEM106B* minor alleles offers strong protection against developing FTLD in *GRN* mutation carriers and argue that TMEM106B functionally interacts with PGRN in the endolysosomal system to affect pathogenesis of FTLD-TDP. A few initial studies showed that the *TMEM106B* protective minor alleles were associated with increased plasma PGRN levels [62, 63], suggesting a potential protective mechanism. However, the increase does not appear to be robust enough to explain the strong protective effect of the minor alleles on FTLD. In addition, studies have failed to replicate the plasma PGRN effect of the *TMEM106B* variation [67, 68]. Therefore, it is likely that TMEM106B also participates in other disease mechanisms associated with PGRN.

Beyond FTLD-*GRN*, *TMEM106B* variation has also been linked to FTLD-TDP with *C9orf72* repeat expansions [69, 70] and many other neurodegenerative diseases including AD [32, 71, 72], chronic traumatic encephalopathy [73], and LATE-NC [60]. In addition, a genetic study has found that *TMEM106B* is a genetic modifier of cognitive decline in PD [74] and of cognitive and motor functions in ALS [75, 76]. Furthermore, a dominant mutation (D252N) in T*MEM106B* was found in a patient with hypomyelinating leukodystrophy (HLD), a group of genetic disorders that affect the development of the myelin sheath in the brain [77], suggesting a role of TMEM106B in myelination.

Multiple SNPs near and in the *TMEM106B* gene, including the SNPs associated with neurodegenerative diseases described above, such as rs1990622, rs6966915, and rs1020004, are in strong linkage disequilibrium (LD), constituting two common *TMEM106B* haplotypes, one associated with increased disease risk, and the other with a protective effect [10, 78] (Fig. 2a). It is therefore difficult to determine the functional variant(s) on the haplotype that are responsible for modulating the disease risk. Within the haplotype block, rs3173615 is the only coding SNP, where threonine at amino acid position 185 on the risk haplotype is changed to serine on the protective haplotype (T185S). To date, cell culture and mouse studies to examine the effects of the amino acid substitution have yielded inconsistent results. We will discuss the T185S variant below in the sections “Endolysosomal functions of TMEM106B” and “Mouse models of PGRN- and/or TMEM106B-associated neurological diseases”. Another study has proposed that the noncoding rs1990620 risk variant increases *TMEM106B* mRNA expression by preferentially recruiting chromatin-organizing protein CCCTC-binding factor (CTCF) and thereby altering chromatin architecture [79]. However, while an independent study has confirmed an increase in *TMEM106B* mRNA in the frontal cortex of individuals with the risk haplotype [7], others have found no significant change [63, 80, 81]. Alternatively, recent studies have identified an AluYb8 element insertion in 3’UTR of the risk haplotype that may act as a functional variant [68, 82] (Fig. 2a). By using publicly available databases as well as a novel large dataset, one of the studies has also shown that the risk haplotype is associated with increased TMEM106B protein levels, but not with *TMEM106B* mRNA expression [68]. Consistent with this result, a proteomic study has reported an age-dependent increase in hippocampal TMEM106B protein levels specifically in the risk variant carriers [83]. Thus, it is possible that the AluYb8 insertion in the risk haplotype causes an increase in TMEM106B protein levels to affect the development and presentation of neurodegenerative diseases.

## PGRN and TMEM106B in brain aging

Aging is a major risk factor for most neurodegenerative diseases [84]. Interestingly, human genetic studies have also linked PGRN and TMEM106B to brain aging. A GWAS study identified both the *GRN* and *TMEM106B* genes as genetic modifiers of biological aging in the human cerebral cortex [85]. In addition, *GRN* and *TMEM106B* variants were shown to interact genetically in the regulation of the biological aging [85]. In this study, *TMEM106B* variants were also found to be associated with inflammation and cognitive deficits even without known brain diseases [85]. Consistent with these results, another study has shown that the *TMEM106B* rs1990621 protective variant is associated with increased neuronal cell proportion specifically in elderly individuals, independent of disease status [86]. Moreover, a recent study finds that the *GRN* and *TMEM106B* AD protective alleles are strongly enriched in cognitively healthy centenarians [87]. These studies highlight the importance of PGRN and TMEM106B in maintained cognitive and brain health during aging.

The critical role of PGRN and TMEM106B in brain aging is also supported by mouse genetic studies, where deletion of either *Grn* or *Tmem106b* gene is reported to cause accelerated accumulation of lipofuscin in the brain [88, 89]. The accumulation of lipofuscin within postmitotic cells like neurons is considered a hallmark of aging because the rate of lipofuscin accumulation positively correlates with that of aging, although it is also associated with some pathological conditions such as lysosomal storage disorders [90, 91]. Importantly, studies have also shown that deleting both *Grn* and *Tmem106b* genes in mice further exacerbates lipofuscin accumulation in the brain [92, 93].

The precise mechanism by which *GRN* and *TMEM106B* variants affect brain aging and whether the relevant pathways also contribute to an increased risk for neurodegenerative diseases are currently unclear. However, given that one of the nine hallmarks of aging is loss of proteostasis [84, 94], it is likely that endolysosomal function of PGRN and TMEM106B plays an important role in brain aging.

## Endolysosomal functions of PGRN

PGRN, encoded by the *GRN* gene in humans, is a 593 amino-acid highly glycosylated protein composed of N-terminal signal sequence followed by 7.5 cysteine-rich granulin domains that are connected by short linker regions [95, 96] (Fig. 1b). PGRN is mainly expressed in neurons and microglia in the brain [97–101], localized to the lysosome, and also secreted into the extracellular space. PGRN expression is regulated by the transcriptional factor EB (TFEB), a major regulator of lysosomal biogenesis and autophagy [102, 103]. There are two TFEB binding sites upstream of the *GRN* coding sequence [102]. PGRN secreted into the extracellular space is delivered to lysosomes by sortilin- or prosaposin (PSAP)-dependent endocytosis in neurons [104–106] (Fig. 1c). In addition, a recent study suggests the existence of additional sortilin and PSAP independent pathways mediating lysosomal trafficking of PGRN in microglia [105].

PGRN can be processed into mature granulins both extracellularly and intracellularly [95]. However, blocking lysosomal delivery of PGRN by knocking out sortilin significantly increases PGRN and decreases granulin levels in mouse brain lysates, suggesting that the PGRN processing occurs predominantly in the lysosome in the mouse brain [104, 105] (Fig. 1c). Several cathepsins and asparagine endopeptidase (AEP) have been proposed to mediate the lysosomal processing of PGRN into granulins [107–110]. Both PGRN and granulins are thought to play a critical role in lysosomal function [95, 96], although the exact functions of individual granulins versus PGRN remain unclear.

PGRN/granulins have been reported to bind to several endolysosomal proteins (Table 1). As described above, PGRN binds to sortilin and PSAP for its lysosomal delivery [104, 106] (Fig. 1c). Sortilin is a type I transmembrane protein of the vacuolar protein sorting 10 (VPS10) family localized to the cell surface, secretory, and endocytic compartments [111]. Sortilin functions as a cell surface PGRN receptor and regulates plasma, serum, and brain PGRN levels [104, 112]. A significant increase in PGRN levels has been reported in the brain and serum of sortilin knockout (KO) mice [104, 105]. PGRN binds to the β-propeller domain of sortilin through its C-terminal tail. It has been shown that deletion of the last three residues of PGRN (QLL) abolishes PGRN binding to sortilin [113]. PSAP is the precursor of saposin peptides that is, like PGRN, secreted into the extracellular space but is also localized or delivered to the lysosome via the PSAP receptors cation-independent mannose 6-phosphate receptor (M6PR) and low-density lipoprotein receptor-related protein 1 (LRP1) [106]. PGRN binds to PSAP through the linker region between saposins B and C [114], and can be delivered to the lysosome via M6PR and LRP1, independent of sortilin [106]. On the other hand, the PGRN-PSAP complex may also be important to facilitate neuronal uptake and lysosomal delivery of extracellular PSAP via sortilin. In the lysosome, PSAP can be processed into saposin peptides (A, B, C, and D), which are known to be involved in lysosomal glycosphingolipid degradation [115].

**Table 1:** Endolysosomal PGRN or TMEM106B-binding proteins with known functional interaction

Binding proteins	PGRN or TMEM106B	Procedures	Functions	References
Cathepsin D	PGRN	Co-IP (cell culture)	Stabilize and activate cathepsin D	[118–121]
Cathepsin D	TMEM106B	Brain IP-MS, co-IP (cell culture)	Stabilize cathepsin D	[132, 138]
Sortilin	PGRN	In vitro SPR, co-IP (cell culture), cell-based binding assay	Accelerate lysosomal delivery	[104, 113]
PSAP	PGRN	Co-IP (cell culture)	Accelerate lysosomal delivery	[106, 114]
GCase	PGRN	In vitro SPR, co-IP (cell culture)	Stabilize and activate GCase	[49, 206–208]
HexA	PGRN	Co-IP (cell culture)	Increase HexA activity	[219]
Rab2	PGRN	Co-IP (cell culture)	Mediate autophagosome-lysosome fusion	[260]
CD68	PGRN	Co-IP (cell culture), cell-based binding assay	Reciprocally stabilize their proteins	[261]
MAP6	TMEM106B	Brain IP-MS, co-IP (Brain and cell culture)	Control dendritic trafficking of lysosomes in neurons	[146]
CHMP2B	TMEM106B	Co-IP (cell culture)	Regulate autophagic flux	[150]
V-ATPase AP1	TMEM106B	Co-IP (cell culture)	Stabilize V-ATPase and lysosomal pH	[132, 137]
GALC	TMEM106B	Brain IP-MS, co-IP (cell culture)	Regulate GALC activity	[138]

Abbreviations: SPR, surface plasmon resonance; IP-MS, immunoprecipitation-mass spectrometry

The other well-characterized PGRN/granulin-binding proteins in the endolysosomal system are β-glucocerebrosidase (GCase) and cathepsin D. We will discuss the role of PGRN-GCase interaction below in the section of “PGRN and TMEM106B in brain lipid metabolism”. Cathepsin D is a lysosomal aspartyl protease that has been linked to several neurodegenerative diseases, including AD and PD [116]. Like PGRN, homozygous mutations in the cathepsin D gene (*CTSD*) cause neuronal ceroid lipofuscinosis (CLN10) [117]. Several groups have independently reported that PGRN or several granulins can bind to cathepsin D [118–121], although the functional consequence of this interaction is still controversial. In vitro, recombinant PGRN and granulins have been shown to stimulate maturation of pro-cathepsin D and/or increase cathepsin D activity [118–120, 122]. Consistent with the in vitro results, reduced cathepsin D activity has been seen in fibroblasts and induced pluripotent stem cell (iPSC)-derived neurons generated from FTLD-*GRN* patients [119, 123]. However, in the mouse brain, PGRN deficiency has been consistently shown to cause an age-dependent increase in both pro- and mature-cathepsin D levels and cathepsin D activity [118, 124–128],, although some studies have reported that cathepsin D activity is decreased in PGRN KO brains when it is normalized to mature cathepsin D protein levels [118, 128]. Another study has shown that, at 2 months of age, cathepsin D activity is not changed in the brain, but decreased in the liver and spleen of PGRN KO mice [121]. Thus, the net effect of PGRN on cathepsin D function remains uncertain.

## Endolysosomal functions of TMEM106B

TMEM106B is a 274 amino-acid highly glycosylated type II transmembrane protein localized to the endolysosomal system [10, 78] (Fig. 2b). TMEM106B is ubiquitously expressed in multiple tissues and in both neurons and glia in the central nervous system [129–133]. TMEM106B is known to form homodimers, which can be detected either by co-immunoprecipitation (co-IP) experiments [134, 135] or by SDS-PAGE under non-reducing conditions [136–138], although the functional importance of the dimers remains unknown. N-terminal cytoplasmic region of TMEM106B is intrinsically disordered without well-defined three-dimensional structure [139], but TMEM106B may undergo N-myristoylation [140–142]. In addition, a study has shown that the extended dileucine-based motif, ENQLVALI, in the cytoplasmic region is a potential lysosomal sorting signal of TMEM106B [143]. In contrast to the disordered N-terminal region, the crystal structure of C-terminal luminal region has revealed a compact fibronectin type III domain with a ubiquitous 7-bladed β sandwich fold, which is closely related to immunoglobulin-like domains [144]. The C-terminal luminal region contains five N-glycosylation sites with the consensus sequence motif N-X-S/T at N145, N151, N164, N183, and N256 [145]. A cell culture study using the glycosylation site mutants has shown that endoglycosidase H-resistant complex glycosylation occurs at N183 and N256 and the complex glycosylation is required for proper transport of TMEM106B to late endosomal/lysosomal compartments [145].

Given its endolysosomal localization, the role of TMEM106B in lysosomal functions has been investigated in numerous cell culture studies. Several studies have consistently shown that overexpression of TMEM106B induces enlargement of lysosomal compartments and formation of LAMP1-positive large cytoplasmic vacuolar structures, while its knockdown results in reduction in the size of lysosomes in immortalized cells and primary neurons [134–136, 143]. TMEM106B levels also affects motility of lysosomes in primary neurons. In DIV20 mouse cortical neurons, overexpression and knockdown of TMEM106B is shown to decrease and increase total lysosomal movements in dendrites, respectively [134]. Other studies have shown that TMEM106B knockdown or KO increases the number of moving lysosomes and retrograde transport of lysosomes in dendrites of DIV9 primary rat hippocampal neurons [146] and in axons of DIV7 primary mouse motoneurons [89]. Maintaining acidic pH (4.2–5.3) is essential for regulating many functions of lysosomes [147]. However, in primary mouse cortical neurons, TMEM106B deficiency has been shown to cause impairment in lysosomal acidification [137], although TMEM106B overexpression has mixed results with some showing an impairment in lysosomal acidification in HeLa cells [136, 143] and others reporting enhanced acidification in HEK293T cells and murine and human lung cancer cell lines [132, 148]. It remains to be determined whether the mixed results stem from the different cell lines used or the different level of overexpression. Although TMEM106B is expressed in both neurons and glia, there are only a few cell culture studies investigating its role in glia. One study using the Oli-Neu oligodendrocyte cell line has suggested that TMEM106B regulates lysosomal function and positioning and PLP trafficking in oligodendrocytes [132]. Another study has shown that TMEM106B deficiency decreases TREM2 levels and cell viability and increases inflammatory responses in cultured microglia [133].

Because several studies demonstrated an association of the *TMEM106B* protective variants with increased human plasma PGRN levels [62, 63], the effect of TMEM106B expression on PGRN levels has been assessed in cell culture studies. Although it is consistently shown that TMEM106B knockdown has no significant effects on PGRN mRNA and protein levels [135, 145, 146], overexpression of TMEM106B has been reported to increase extracellular and/or intracellular PGRN levels [61, 135, 136] and reduce PGRN processing into granulins [108] in certain cell lines. Overexpression of TMEM106B has been reported to induce nuclear translocation of TFEB and expression of TFEB-regulated lysosomal genes in HEK293 cells, primary cortical neurons, and lung cancer cells [134, 148]. However, an increase in PGRN mRNA by TMEM106B overexpression has not been reported in other studies [135, 145]. It remains unclear whether the results from these cell culture studies are responsible for the human observations since the *TMEM106B* protective variants may reduce TMEM106B protein levels [7, 10, 68, 78, 79, 83].

Several studies have explored TMEM106B-binding proteins (Table 1). A yeast two-hybrid (Y2H) screen using cytoplasmic or luminal fragment of TMEM106B for bait and a prey library derived from human adult brain has identified TMEM106C, the endocytic adaptor proteins, AP2M1 and CLTC, and the vacuolar protein sorting proteins, VPS11 and VPS13D as N-terminal TMEM106B-binding proteins, although the functional importance of these interactions remains to be determined [134]. In the Y2H screen, no proteins have been found to bind to C-terminal TMEM106B [134]. An immunoprecipitation (IP) and liquid chromatography tandem mass spectrometry (LC-MS/MS) analysis using P15 rat brain has identified microtubule-associated protein 6 (MAP6) as a TMEM106B-binding protein [146]. The TMEM106B-MAP6 interaction has been confirmed by co-IP experiments and shown to be crucial for controlling dendritic trafficking of lysosomes in primary rat neurons [146]. Another IP and LC-MS/MS analysis using WT and TMEM106B KO mouse brains has identified 22 potential lysosomal TMEM106B-binding proteins, including vacuolar-ATPase (V-ATPase), cathepsin D, and galactosylceramidase (GALC) [138]. Co-IP experiments have confirmed the TMEM106B-V-ATPase, especially V-ATPase accessary protein 1 (AP1) [132, 137], TMEM106B-cathepsin D [132, 138], and TMEM106B-GALC interactions [138]. V-ATPase is an ATP-driven proton pump involved in acidification of intracellular compartments [149]. TMEM106B deficiency has been shown to cause down-regulation of several V-ATPase V0 subunits and AP1 in the mouse brain and impair lysosomal acidification in primary mouse neurons [137]. The TMEM106B-cathespin D interaction may be important for maintaining proper cathepsin D levels [132]. We will discuss the role of TMEM106B-GALC interaction in the section of “PGRN and TMEM106B in lipid metabolism” below. A co-IP experiment using HEK293T cells has shown association between TMEM106B and charged multivesicular body protein 2B (CHMP2B), a subunit of the endosomal sorting complex required for transport-III (ESCRT-III) [150]. A mutation in *CHMP2B* has been reported to causes an autosomal dominant form of frontotemporal dementia [151]. TMEM106B-CHMP2B interaction may affect the ESCRT-mediated endosomal and autophagy pathways [150].

As aforementioned, T185S variant and D252N mutation of TMEM106B have been associated with FTLD and HLD, respectively. Multiple cell culture studies have assessed the effects of these amino acid substitutions on TMEM106B functions. First, the T185S variant and D252N mutation are located near the complex glycosylation sites N183 and N256. However, no significant effects of these amino acid substitutions on glycosylation have been reported so far [61, 132]. Studies have also shown that T185S variation has no significant effects on homodimerization of TMEM106B [135], on TMEM106B-GALC interaction [138], or on TMEM106B-induced lysosomal enlargement [135], nuclear translocation of TFEB [134], or increases of PGRN [61, 135]. An overexpression study has shown that T185S variation destabilizes TMEM106B protein [61], but this is not supported by other cell culture and mouse studies [135, 150, 152, 153]. Another study has reported that T185S variation significantly reduces association of TMEM106B with wild-type and mutant CHMP2B^Intron5^ and abolishes exacerbating effects of TMEM106B on CHMP2B^Intron5^-induced endosomal/autophagic defects and neurotoxicity [150]. For HLD-associated D252N mutation, studies have shown no significant effects of the mutation on the dimerization [132], TMEM106B-V-ATPase AP1 interaction [132], TMEM106B-cathepsin D interaction [132], and TMEM106B-GALC interaction [138]. However, D252N mutation has been reported to abolish TMEM106B-induced lysosomal enlargement and acidification [132]. In addition, a study using patient-derived fibroblasts has shown that heterozygous D252N mutation causes lysosomal dysfunction [154].

## Amyloid fibrils of a C-terminal fragment (CTF) of TMEM106B

Recent studies using cryo-electron microscopy (cryo-EM) discovered amyloid fibrils composed of TMEM106B CTF corresponding to residues 120–254 from the brains of aged people ( > ~ 45 years) without known neurological disease as well as subjects with FTLD-TDP and a variety of other neurodegenerative diseases including AD, PD, LBD, progressive supranuclear palsy (PSP), corticobasal degeneration (CBD), and ALS [155–161]. Immunohistochemical analysis using antibodies against the luminal domain of TMEM106B has shown that TMEM106B aggregates are found in both neurons and glia and are most abundant in brain astrocytes [129, 162, 163]. In addition, greater TMEM106B pathology has been observed in FTLD-*GRN* and LATE-NC cases [160, 163]. Immunoblot analysis has shown that the *TMEM106B* risk haplotype is associated with increased sarkosyl-insoluble 29 kDa CTF of TMEM106B [83, 164, 165], while decreasing dimeric TMEM106B in the RIPA-soluble fraction [164].

Although TMEM106B reportedly undergoes physiological processing [166, 167], the exact mechanism and proteases involved in the C-terminal cleavage of TMEM106B to form the fibrils are currently unclear. It appears that a precise processing of TMEM106B between resides 119 and 120 is required for the amyloid fibril formation because cryo-EM structure showed that Ser120 is buried in the fibril core 120–254, leaving no space for the other N-terminal residues [158]. In addition, there may be a second cleavage at some residue after Gly254 since antibodies against the extreme C-terminus (the residues 263–274, 253–274, and/or 259–274) of TMEM106B did not detect TMEM106B inclusions by immunostaining [129, 162] or sarkosyl-insoluble 29 kDa CTF by immunoblot [167] (Fig. 2c). A recent study has shown that the C-terminal trimming also occurs under physiological conditions [167]. Three major filament folds and two doublet polymorphisms have been described, although unlike other amyloid proteins such as tau, a-synuclein, and TDP-43, no relationship between the filament fold and disease was observed [156, 158] (Fig. 2d). So far, the TMEM106B fibrils have been identified only from human tissues and have not been found from any mouse tissues [129] or in cell culture systems. It is notable that a recent study has found amyloid fibrils of TMEM106B CTF in Biondi bodies, filamentous amyloid inclusions in ependymal cells of the choroid plexuses in aged human brains [168]. Biondi bodies have not been found in nonhuman mammals except for chimpanzees [169]. The result raises the possibility that TMEM106B fibrils may be formed only in certain primates.

It also remains unclear whether the TMEM106B fibrils play a role in brain aging and/or pathophysiology of neurodegenerative diseases. Recently, TMEM106B CTFs were shown to aggregate and cause neuronal loss and behavioral deficits when expressed in neurons of *C. elegans* [170]. However, the experimental system included N-terminal dendra2-tagged CTF, which likely forms aggregates with a structure distinct from ones observed in human brains. Therefore, the results need to be cautiously interpreted.

## Mouse models of PGRN- and/or TMEM106B-associated neurological diseases

While PGRN haploinsufficiency causes FTLD-TDP, *Grn* heterozygous null (*Grn*^+/−^) mice develop no obvious disease-associated pathologies, such as TDP-43 inclusions, in the brain. However, some studies using a large cohort of mice have reported subtle but significant lysosomal dysregulation and behavioral abnormalities such as social deficits in *Grn*^+/−^ mice, which may be relevant to FTLD-*GRN* [98, 171, 172]. In addition, a recent study has shown that *Grn*^+/−^ mice have reduced dendritic spine head diameter in the prefrontal cortex [173].

While *Grn* homozygous null (*Grn*^−/−^) mice are viable and fertile, they exhibit several features of FTLD-*GRN* and CLN11 including lysosomal dysregulation, lipofuscinosis, neuroinflammation, synaptic loss, retinal ganglion cell (RGC) loss, and obsessive compulsive disorder (OCD)-like behavior as well as social deficits [16, 88, 137, 174–177] and therefore have been widely used as a model of FTLD-*GRN* and CLN11. However, *Grn*^−/−^ mice fail to develop neurodegeneration or TDP-43 pathology in the cerebral cortex, although several studies have described selective loss of excitatory neurons and cytoplasmic TDP-43-positive aggregates in the thalamus of aged *Grn*^−/−^ mice [175, 176, 178]. *Grn*^*R493X*^ knockin mice harboring one of the most common human *GRN* mutations, a premature termination codon (PTC) at arginine 493 (R493X), have also been generated [179]. Homozygous *Grn*^*R493X*^ knockin mice have significantly reduced *Grn* mRNA levels due to nonsense-mediated mRNA decay (NMD) and do not express detectable PGRN protein, resulting in a phenocopy of *Grn*^−/−^ mice [179–181]. Thus, *Grn*^*R493X*^ knockin mice may be a useful model to test therapeutic approaches targeting PTC or NMD (Table 2).

**Table 2:** Mouse models of PGRN- and/or TMEM106B-associated neurological diseases

Mouse models	Diseases	Behavioral phenotypes	Histological phenotypes	Biochemical phenotypes	References
*Grn*^*+/−*^ mice	FTLD	Social deficits	Lysosomal dysfunction	Lysosomal dysfunction	[98, 171, 172]
*Grn*^*−/−*^ mice	FTLD, CLN11	Social deficits, OCD-like behavior	Lysosomal dysfunction, lipofuscinosis, neuroinflammation, complement activation, RGC loss, inhibitory synaptic and excitatory neuronal loss and Increased cytoplasmic TDP-43 in the thalamus	Lysosomal enzyme dysregulation, decreased BMP, increased GlcCer/Sph and gangliosides	[16, 88, 137, 174–178]
*Grn*^*R493X/R493X*^ mice	FTLD, CLN11	Social deficits, OCD-like behavior	Lysosomal dysfunction, lipofuscinosis, neuroinflammation, inhibitory synaptic and excitatory neuronal loss in the thalamus	Lysosomal enzyme dysregulation, decreased BMP, increased GlcSph and gangliosides	[179–181]
*Tmem106b*^*−/−*^ mice	FTLD, HLD	Late-onset motor deficits	Minor myelination deficits, vacuole accumulation, late-onset cerebellar gliosis and Purkinje cell loss	Lysosomal enzyme dysregulation	[89, 132, 133, 187–190]
*Grn*^*−/−*^ *Tmem106b* Tg mice^a^	FTLD	No data available	Exacerbated lysosomal abnormalities and lipofuscinosis	No data available	[191]
*Grn*^*+/−*^ *Tmem106b*^*+/−*^ mice	FTLD	No amelioration of *Grn*^*+/−*^ phenotypes	No data available	No amelioration of *Grn*^*+/−*^ phenotypes	[192]
*Grn*^*−/−*^ *Tmem106b*^*+/−*^ mice	FTLD	No data available	No amelioration of *Grn*^*−/−*^ phenotypes	No amelioration of *Grn*^*−/−*^ phenotypes	[193]
*Grn*^*−/−*^ *Tmem106b*^*H/H*^ mice^b^	FTLD	Improved disinhibition, but late-onset motor deficits	Attenuated lysosomal enzyme dysfunction, reduced RGC loss, no amelioration of microglial activation and lipofuscin	Attenuated lysosomal enzyme dysregulation	[137, 194, 195]
*Grn*^*−/−*^ *Tmem106b*^*−/−*^ mice	FTLD	Early-onset motor deficits	Exacerbation of *Grn*^*−/−*^ phenotypes, neurodegeneration and TDP-43 pathology in the brainstem and spinal cord.	Exacerbation of *Grn*^*−/−*^ phenotypes, more insoluble p-TDP-43 in the brainstem and spinal cord	[92, 93, 193, 195]
*Grn*^*−/−*^ *Tmem106b*^*T186S/T186S*^ mice	FTLD	No data available	No amelioration of *Grn*^*−/−*^ phenotypes	No amelioration of *Grn*^*−/−*^ phenotypes	[153]
APPswe/PS1ΔE9 *Grn*^*−/−*^ mice	AD	Improved learning deficit	Reduced diffuse Aβ plaque burden, more activated microglia near plaques, more complement deposition	Reduced insoluble Aβ	[52]
5XFAD *Grn*^*−/−*^ mice	AD	No obvious behavioral changes in the open-field and Y-maze	Reduced Aβ pathology in male, more activated microglia near plaques, increased expression of lysosomal proteins	Reduced APP and Aβ in young male, increased expression of lysosomal proteins	[182]
Tg2576 *Grn*^*+/−*^ mice	AD	No data available	Reduced Aβ deposition	Reduced insoluble Aβ	[183]
J20 *Grn*^*flox/flox*^ LysM-Cre	AD	exacerbated learning and memory deficits	Increased hippocampal Aβ deposition	No data available	[184]
JNPL3 *Grn*^*+/−*^ mice	Tauopathy	No data available	More p-tau inclusions	Increased soluble and insoluble p-tau	[185]
PS19 *Grn*^*−/−*^ mice	Tauopathy	Exacerbated disinhibition, but improved memory deficits	More GlcCer-positive tau and α-synuclein inclusions, but attenuated hippocampal atrophy	No change in insoluble tau, higher GlcCer	[49]
PS19 *Tmem106b*^*−/−*^ mice	Tauopathy	Hyperactivity, exacerbated motor and cognitive deficits	Exacerbated tau pathology, neuroinflammation, and neurodegeneration	Increased soluble and insoluble p-tau	[152, 198]
PS19 *Tmem106b*^*T186S/T186S*^ mice	Tauopathy	Improved cognitive decline	Attenuated hippocampal atrophy, no changes in glial and tau pathology	No change in p-tau	[152]

^a^ *Tmem106b* Tg, transgenic mice with elevated TMEM106B levels

^b^ *Tmem106b*^*H/H*^, mice with homozygous *Tmem106b* hypomorphic alleles

To examine the role of neuronal versus microglial PGRN, several conditional PGRN KO mice have been generated. Studies using *Grn*^*flox/flox*^ mice crossed with CaMKII-Cre, Nestin-Cre, or LyzM-Cre have demonstrated that neither neuronal nor microglial loss of PGRN is sufficient to induce lipofuscinosis and neuroinflammation in mice [97–99]. In addition, mice with depletion of both neuronal and microglial PGRN have also failed to develop lipofuscinosis and gliosis [101]. Therefore, these phenotypes appear to require essentially complete loss of PGRN in mice. Notably, two neuronal PGRN deficient mouse lines, CaMKII-Cre *Grn*^*flox/flox*^ mice and Nestin-Cre *Grn*^*flox/flox*^ mice, have been shown to develop social deficits, highlighting an important role for neuron-derived PGRN in social function [98]. In addition, microglial PGRN deficient mouse lines, LyzM-Cre *Grn*^*flox/flox*^ mice and tamoxifen-treated Cx3Cr1-Cre^ERT2^ *Grn*^*flox/flox*^ mice, exhibit excessive grooming, an OCD-like behavior, which is also observed in constitutive *Grn*^−/−^ mice [100, 101].

To investigate the role of PGRN in AD and the mechanism by which *GRN* variants and mutations increase AD risk, numerous AD mouse models have been analyzed on PGRN-deficient background (Table 2). Studies using APPswe/PS1∆E9 and 5XFAD mouse models of amyloid pathology have shown that constitutive loss of PGRN has no exacerbating effects on Aβ pathology [52, 182], which is consistent with human ADNI biomarker data showing no effects of the *GRN* rs5848 AD variant on amyloid pathology [52]. In fact, a reduction of Aβ pathology has been reported in those mice with PGRN loss. In addition, a study has shown that PGRN haploinsufficiency reduces amyloid pathology in female Tg2576 APP transgenic mice although only a limited number of mice were examined [183]. In contrast, selective reduction of microglial PGRN by crossing with LysM-Cre mice has been shown to significantly increase Aβ pathology in J20 APP transgenic *Grn*^*flox/flox*^ mice [184]. It remains unclear whether the contrasting results stem from differences in PGRN manipulation or simply from different AD mouse models utilized in those studies. The role of PGRN in tau pathology, another hallmark of AD, has also been investigated using several mouse models of tauopathy. Interestingly, not only complete loss but also haploinsufficiency of PGRN has been shown to increase tau pathology in P301L (JNPL3) and P301S (PS19) mouse models of tauopathy [49, 185], although tau-mediated neurodegeneration was paradoxically attenuated in PGRN-deficient PS19 tauopathy mice [49]. PGRN deficiency has been reported to have no significant effects on tau spreading induced by AD-derived tau fibrils in the mouse brain [186].

TMEM106B homozygous KO (*Tmem106b*^−/−^) mice are viable and fertile and exhibit no overt behavioral deficits during developmental and adult stages, but show minor myelination deficits in histopathological analysis [132, 187], consistent with human evidence that a *de novo* mutation in *TMEM106B* causes HLD [77]. The histological deficits include decreases in MBP, PLP, MOG, or OLIG2 immunostaining and Black Gold II stain in the corpus callosum [132, 133, 187]. Transcriptomic analysis has also shown global changes in myelin pathway in *Tmem106b*^−/−^ brains [187]. In addition, *Tmem106b*^−/−^ mice are more susceptible to cuprizone-induced demyelination and have a reduced capacity to remyelinate the corpus callosum [133, 187]. In addition to myelination deficits, accumulation of LAMP1-positive vacuoles is observed at the axon initial segment of motoneurons of *Tmem106b*^−/−^ brains [89]. Aged *Tmem106b*^−/−^ mice have been reported to develop motor deficits associated with cerebellar gliosis and Purkinje cell loss [188, 189] (Table 2). It is notable that increased Purkinje cell loss is also observed in human subjects with a *TMEM106B* disease risk allele [190]. To examine a role of TMEM106B in microglia in vivo, microglial TMEM106B deficient mice have been generated by treating Cx3Cr1-Cre^ERT2^ *Tmem106b*^*flox/flox*^ mice with tamoxifen [133]. Microglia-specific loss of TMEM106B resulted in decreased microglial proliferation and activation in response to LPS [133]. Although TMEM106B is expressed in both neurons and glia [130–133], no other cell type-specific deletions of *Tmem106b* have been investigated so far.

Given the role of *TMEM106B* variants in FTLD-*GRN* [7, 61–66], several TMEM106B transgenic and KO mice have been crossbred with PGRN-deficient mice (Table 2). The *TMEM106B* protective/risk variants are predicted to reduce/increase TMEM106B protein without a complete loss [7, 10, 68, 78, 79, 83]. Thus, assessing the effects of partial reduction/increase of TMEM106B levels in *Grn*^+/−^ or *Grn*^−/−^ mice may be the most relevant to understanding human variants. Regarding this point, one study generated transgenic mice expressing human TMEM106B under the neuronal specific CaMKII promoter [191]. In these TMEM106B transgenic mice, total TMEM106B levels were not altered in the brain and were only significantly increased on *Grn*^−/−^ background at 17–20 months of age. However, the elevated TMEM106B has been shown to exacerbate accumulation of lipofuscin and enlarged lysosomes in *Grn*^−/−^ brains [191]. In contrast, TMEM106B haploinsufficiency has reportedly no significant effects on phenotypes of *Grn*^+/−^ [192] and *Grn*^−/−^ mice [193]. Although *Tmem106b*^*T186S*^ knockin mice harboring the disease protective T185S variant have recently also been generated and crossed with *Grn*^−/−^ mice, no protective effects of the T185S variant have been found in *Grn*^−/−^ mice [153]. By immunoblot analysis, no significant changes in TMEM106B protein levels or apparent molecular weight have been observed in the brain of *Tmem106b*^*T186S*^ knockin mice [152, 153], which is inconsistent with the results from the cell culture study described earlier [61]. The first TMEM106B-deficient mouse line reported [137] was generated by a gene trap strategy and found to be a hypomorph expressing 5–10% residual endogenous full-length TMEM106B [194, 195]. Interestingly, this hypomorphic mutation in *Tmem106b* ameliorates lysosomal and behavioral phenotypes of adult but not aged *Grn*^−/−^ mice [137, 194, 195].

Several TMEM106B complete null mouse lines have also been crossbred with PGRN-deficient mice [92, 93]. Importantly, in contrast to the hypomorphic mutation described above, complete loss of TMEM106B on *Grn*^−/−^ background leads to severe early motor dysfunction in mice. The double KO (DKO) mice also exhibit robust lysosomal and FTLD-*GRN*-like pathologies including neurodegeneration and TDP-43 inclusions in the brainstem and spinal cord [92, 93, 193]. Therefore, this DKO line has been proposed as a mouse model of FTLD-*GRN* and ALS/FTLD [196, 197], since single *Grn*^−/−^ mice do not show robust neuronal loss or TDP-43 pathology as described above. However, it is worth noting that the *TMEM106B* risk variants do not cause complete loss of TMEM106B protein and previous studies have suggested that the risk variants may rather increase TMEM106B levels [7, 10, 68, 78, 79, 83]. Therefore, use of the DKO line as a model of FTLD-*GRN* with *TMEM106B* risk variants must be interpreted cautiously. Taken together with the hypophorphic allele results, the data suggest that TMEM106B levels have biphasic effects, reducing *Grn*^−/−^ pathology with 90% reduction but exacerbating *Grn*^−/−^ pathology with 100% TMEM106B reduction.

The role of TMEM106B in tauopathy has been also explored in mouse models. Recent studies have shown that complete loss of TMEM106B exacerbates tau pathology and tau-mediated neurodegeneration in the PS19 tauopathy mouse model [152, 198], while the T185S protective variant protected against cognitive deficits and neurodegeneration without affecting tau pathology [152] (Table 2). These results may be consistent with human genetic studies showing a link between *TMEM106B* variants and increased AD risk [32, 71, 72], although the underlying mechanism remains unknown. Effects of TMEM106B deficiency on tau spreading has been investigated in an AD-tau injection mouse model. However, similar to the results with *Grn*^−/−^ mice, no significant effects of TMEM106B loss on tau spreading have been found [186]. To date, effects of TMEM106B deficiency on Aβ pathology and Aβ-associated deficits has not been reported for preclinical models.

Most recently, a novel transgenic mouse model expressing human TMEM106B under the CAG promotor was generated [199]. In contrast to the prior model described above [191], the transgenic mice successfully overexpress human TMEM106B, with a 4- to 8-fold increase in the protein levels in hemizygotes and homozygotes, respectively, as compared to the levels of endogenous mouse TMEM106B. Interestingly, down-regulation of immediate early genes, altered synaptic signaling, an anxiety-like behavior, and mild neuronal loss have been observed in these transgenic mice, suggesting that increased TMEM106B levels negatively impact brain health [199]. The transgenic mice have not been crossbred with PGRN-deficient mice or other mouse models of neurodegenerative diseases yet.

## Human iPSC models of FTLD-*GRN* and CLN11

The differential effects of PGRN haploinsufficiency on human and mouse brains and difficulty of reproducing effects of the *TMEM106B* risk haplotype in mouse models may suggest the existence of human-specific pathogenic mechanisms in FTLD-*GRN*, and experimental systems using human patient-derived iPSC lines may be useful to explore such mechanisms. However, there are currently only a few published studies investigating in vitro models using iPSCs with heterozygous *GRN* mutations. So far, none of these studies show clear TDP-43 inclusions by immunostaining. Two studies using iPSC lines generated from FTLD-*GRN* patients carrying heterozygous S116X nonsense mutation and A9D missense mutation have observed PGRN haploinsufficiency and cytoplasmic mis-localization of TDP-43 in iPSC-derived neurons [119, 200]. The iPSC-derived neurons with *GRN* A9D mutation have also shown accumulation of insoluble TDP-43 and lipofuscin and reduced cathepsin D activity as mentioned earlier [119]. Another study using an iPSC line generated from FTLD patients carrying heterozygous *GRN*^IVS1+5G >C^ mutation did not detect TDP-43 aggregation, but has observed insufficient differentiation into cortical neurons, but not motor neurons, which can be rescued by genetic correction or WNT inhibitor [201]. There are currently no studies examining iPSC lines generated from *TMEM106B* protective versus risk variants.

Although potentially less relevant to FTLD-*GRN*, several previous studies have established in vitro models using iPSC lines with homozygous *GRN* mutation. A recent study has reported that iPSC-derived cortical neurons carrying CLN11-causing *GRN* Thr272fsX10 homozygous mutation display TDP-43 mis-localization and cleavage as well as lipofuscin accumulation [202]. Another study using iPSC-derived neuron/astrocyte co-cultures has shown that *GRN*^*R493X/R493X*^ astrocytes significantly delay spiking activity in developing neurons [203]. In addition, *GRN* homozygous null (*GRN*^−/−^) human mature brain organoids composed of iPSC-derived cortical neurons and astrocytes have been shown to recapitulate TDP-43 mis-localization, hyperphosphorylation, and mis-splicing of *STMN2* transcripts [204], which is an indicator of TDP-43 pathology [205]. Finally, similar to *Grn*^−/−^ *Tmem106b*^−/−^ DKO mice, a study has observed a significant increase in TDP-43 cleavage and phosphorylation, and in cathepsin B, D, and L activities of co-cultures composed of WT iPSC-derived neurons and *GRN*^−/−^ *TMEM106B*^−/−^ DKO iPSC-derived microglia, although the effects of single gene KO were not evaluated [197].

## PGRN and TMEM106B in brain lipid metabolism

**Fig. 3:**
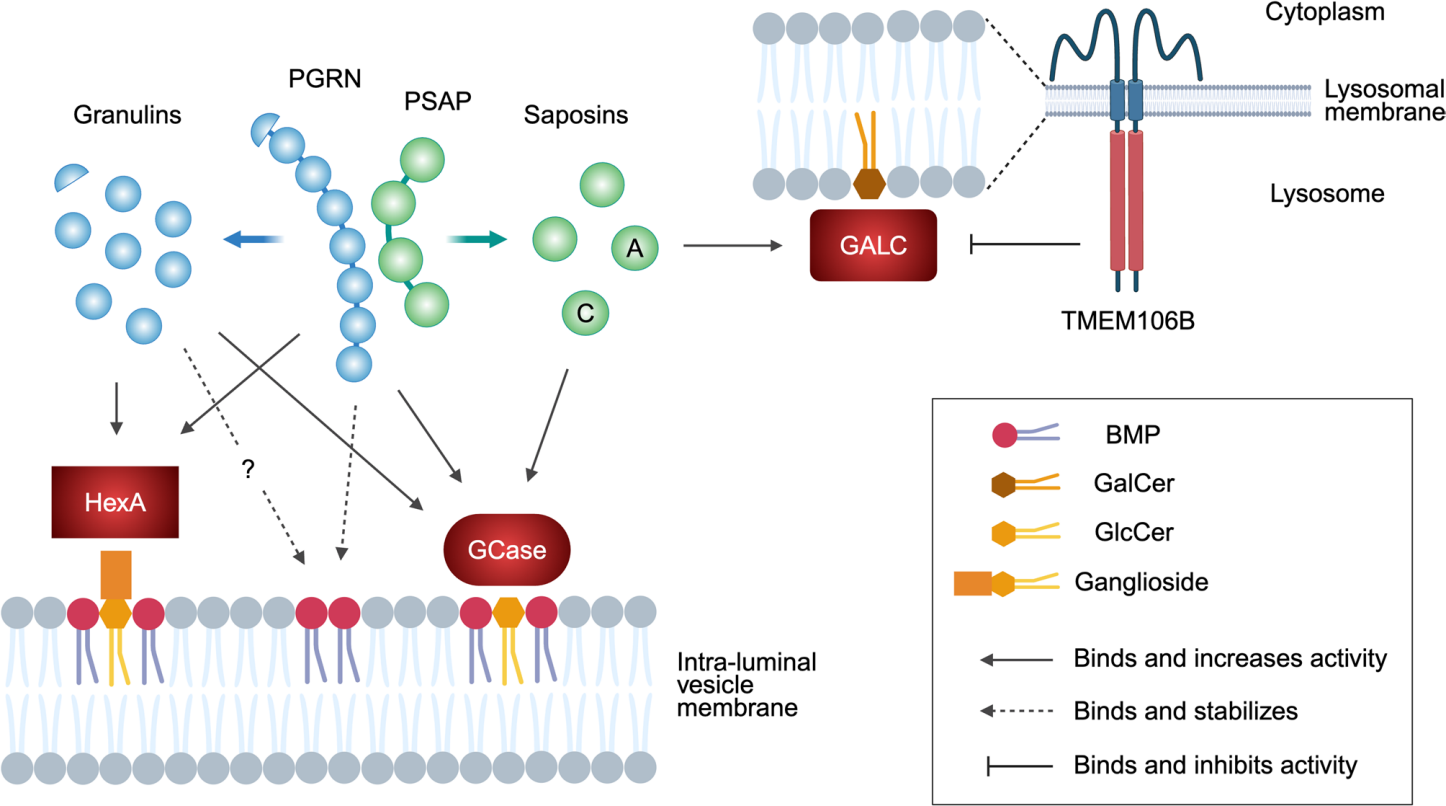
Glycosphingolipid regulation by PGRN and TMEM106B. PGRN and/or granulins bind to GCase and HexA and promote their activity to limit accumulation of GlcCer (and GlcSph) and gangliosides in lysosomes. PGRN also binds and stabilizes anionic phospholipid BMP, which stimulates GCase activity and ganglioside degradation at the intraluminal vesicles of lysosomes. Granulins may also bind and stabilize BMP, although it has not been proven. TMEM106B binds to GALC and regulates its activity to maintain GalCer levels in lysosomes. PSAP is cleaved into saposins A to D in lysosomes and saposins A and C are known to stimulate GALC and GCase activity, respectively. Figure was created with BioRender.com

Several groups have independently shown that PGRN physically interacts with glucocerebrosidase (GCase), a lysosomal enzyme that hydrolyzes the glycosphingolipids glucosylceramide (GlcCer) and glucosylsphingosine (GlcSph) [49, 206–208]. Biallelic mutations in the *GBA1* gene, encoding GCase, result in the lysosomal storage disorder Gaucher disease, while heterozygous mutations have been identified as a common risk factor for PD [209]. PGRN deficiency attenuates GCase activity in the mouse brain [49, 206, 208, 210] and tissues [206]. Reduced GCase activity has also been reported in the brain of FTLD-*GRN* patients [208] as well as iPSC-derived *GRN* mutant neurons [211]. Lipidomic analysis confirms an increase in GlcCer or GlcSph levels in the brain of *Grn*^−/−^ mice [49, 124, 210, 212] and *Grn*^−/−^ *Tmem106b*^−/−^ DKO mice [197]. An increase in GlcSph levels is also observed in the plasma of FTLD-*GRN* patients [210, 213]. Together, these studies suggest that PGRN binds GCase and potentiates the enzymatic activity to limit GlcCer levels in the brain (Fig. 3). PGRN binding to GCase may also stabilize lysosomal localization of GCase [49, 57]. Besides the direct binding to GCase, PGRN has also been suggested to increase GCase activity indirectly by affecting co-factors such as saposin C [211] or bis(monoacylglycero)phosphate (BMP) [210] as described below. It is important to note that a previous study has shown association of *GRN* variants with Gaucher disease [57], demonstrating a genetic interaction between *GRN* and *GBA1*. Consistent with the human genetic study, PGRN deficiency has been shown to exacerbate Gaucher disease-associated phenotypes of *Gba1*^*D409V/D409V*^ knockin mice [214].

Recent lipidomic studies from multiple groups have found a significant and robust decreases in BMP levels in the brain of *Grn*^−/−^ mice [49, 124, 210, 212, 215–217] and *Grn*^−/−^ *Tmem106b*^−/−^ DKO mice [197]. BMP is an anionic phospholipid enriched in the endolysosomal compartment and its intralumenal vesicles [218]. A study has shown that adding liposomes containing BMP restores attenuated GCase activity in PGRN-deficient mouse brain lysates, suggesting that BMP deficiency may underlie GCase activity defects in *Grn*^−/−^ mice [210]. A decrease in BMP levels has also been observed in CSF and the brains of FTLD-TDP patients with and without *GRN* mutations [210, 215], while an increase in BMP has been detected in urine samples of FTLD-*GRN* patients treated with AAV expressing PGRN [216]. The mechanism underlying this positive correlation between PGRN and BMP levels is still not fully understood. In vitro, His-tagged PGRN binds to liposomes containing BMP [210, 215], suggesting a direct effect of PGRN on BMP stability and/or turnover (Fig. 3). However, one study has found that only a minor fraction of untagged PGRN binds to liposomes containing BMP, and a control protein, His-tagged Cas9, also binds to the liposomes [215], suggesting that the His-tag might be responsible. Further investigation is required to elucidate the exact mechanism.

Accumulation of gangliosides, which are sialic acid-containing glycosphingolipids, is also reported in the brain of *Grn*^−/−^ mice [212, 215, 219], *Grn*^−/−^ *Tmem106b*^−/−^ DKO mice [197], and FTLD-*GRN* patients [215]. Similar to GCase activity defects described above, a study has shown that ganglioside accumulation can be rescued by supplementation of BMP in PGRN-deficient HeLa cells [215]. Therefore, the ganglioside accumulation may also be a consequence of BMP deficiency caused by PGRN loss, which is consistent with a previous study demonstrating that BMP stimulates ganglioside degradation [220]. However, there is also a published study demonstrating that PGRN binds and increases the enzymatic activity of β-hexosaminidase A (HexA), a lysosomal enzyme that breaks down GM2 ganglioside [219] (Fig. 3).

In addition to specific lipid classes such as GlcCer and BMP, PGRN may regulate formation of lipid droplets in the cell. Lipid droplets are lipid-storing organelles composed of a neutral lipid core, primarily consisting of triacylglycerols (TAGs) and/or cholesterol esters, surrounded by a phospholipid monolayer [221] and known to accumulate in microglia in the aging brain [222]. Lipid-droplet-accumulating microglia were shown to be defective in phagocytosis and secrete proinflammatory cytokines [222, 223]. A CRISPR-Cas9 screen using BV2 mouse microglial cell line identified *Grn* as a genetic regulator of lipopolysaccharide-induced lipid droplet formation [222]. It was also reported that *Grn*^−/−^ mice contain high numbers of lipid-droplet-rich microglia [222]. Another study has shown an increase in perilipin 2-positive lipid droplets in microglia of cuprizone-treated female *Grn*^−/−^ mice [224]. In addition, PGRN haploinsufficiency has been shown to lead to aberrant lipid droplet formation in human monocyte-derived microglia-like cells [225]. The mechanism by which PGRN deficiency increases lipid droplets in microglia is currently unknown. Although an increase in polyunsaturated TAGs has been reported in *Grn*^−/−^ mouse embryonic fibroblasts and in the liver of *Grn*^−/−^ mice [226], it remains unclear whether TAGs are also increased in *Grn*^−/−^ brains and whether the increase is a consequence or a cause of lipid droplet formation.

TMEM106B has also been implicated in lipid metabolism. A previous study has predicted that TMEM106B is a lipid transfer protein [227]. The luminal region of TMEM106B is found to be a member of late embryogenesis abundant-2 (LEA-2) domain superfamily, which has a long, conserved lipid-binding groove. Interestingly, two identified yeast LEA-2 homologues, Vac7 and Tag1, are localized to the degrative vacuole in yeast (equivalent to the lysosome in humans) and regulate PI(3,5)P_2_ generation and autophagy, respectively [227].

A recent targeted lipidomic analysis using TMEM106B-deficient mice has revealed that TMEM106B deficiency significantly decreases the glycosphingolipids galactosylceramide (GalCer) and its sulfated derivative sulfatide levels in the brain [138]. A decrease in several species of GalCer is also reported in the brain of *Grn*^−/−^ *Tmem106b*^−/−^ DKO mice, which was not rescued by AAV-mediated liver expression of brain-penetrant PGRN biologics [197]. GalCer and sulfatide are two major classes of myelin lipids, which together constitute ~ 27% of the myelin lipid [228]. Thus, the results are consistent with the finding that *TMEM106B* is a causal gene of HLD [77]. In humans, hexosylceramide and sulfatide levels are shown to be significantly lower in the *TMEM106B* disease risk variant carriers [83]. Mechanistically, TMEM106B physically interacts with galactosylceramidase (GALC), a lysosomal enzyme that hydrolyzes GalCer, via its luminal region (Fig. 3). GALC activity, but not protein levels, is significantly increased in TMEM106B-deficient mouse brains [138]. Whether alteration of GalCer and/or sulfatide levels is responsible for TMEM106B-associated neurological diseases such as HLD requires further investigation.

## PGRN-boosting therapies

There are currently no approved therapies for FTLD-*GRN* and CLN11. As FTLD-*GRN* and CLN11 are caused by PGRN deficiency, an effective therapy for these diseases may be to increase PGRN protein levels by gene therapy or protein replacement therapy. Indeed, adeno-associated virus (AAV)-based gene therapy has been successfully used to restore PGRN levels and to improve pre-existing pathologies and deficits in *Grn*^+/−^ and *Grn*^−/−^ mice [98, 128, 216, 229]. In addition, AAV1/9-mediated PGRN expression has been reported to partially prevent brain pathologies and motor deficits of *Grn*^−/−^ *Tmem106b*^−/−^ DKO mice [196]. Interim results from a phase 1/2 clinical trial of AAV-based *GRN* gene therapy (PR006) have demonstrated that one-time injection of PR006 into the cisterna magna is generally safe and well tolerated and increases CSF PGRN levels [216]. In addition, two other AAV-based *GRN* gene therapies, PBFT02 and AVB-101, are currently in phase 1/2 clinical trials (NCT04747431 and NCT06064890, respectively) (Table 3). To avoid the logistics of central nervous system (CNS) injection and its potential safety concerns [230], recent preclinical studies have investigated therapeutic strategies using transferrin receptor (TfR) binding, brain-penetrant PGRN biologics, enabling TfR-mediated transcytosis across the blood-brain barrier (BBB) and enhanced CNS delivery [210, 231]. Peripheral administration and AAV-mediated liver expression of brain-penetrant PGRN biologics have been shown to rescue phenotypes of *Grn*^−/−^ mice and *Grn*^−/−^ *Tmem106b*^−/−^ DKO mice, respectively [197, 210]. A brain-penetrant recombinant PGRN biologic (DNL593) is currently under investigation in a phase 1/2 clinical trial (NCT05262023) (Table 3).

**Table 3:** PGRN-boosting therapies in clinical trials

Modality	Name	Stage	CTGID	Mechanism of action	Route of administration	Result	References
Anti-sortilin antibody	Latozinemab (AL001)	Phase 3	NCT04374136	Block lysosomal delivery of PGRN to increase extracellular PGRN	Intravenous (IV)	Under investigation	[232, 236]
Anti-sortilin antibody	Latozinemab (AL001)	Phase 2	NCT03987295	Block lysosomal delivery of PGRN to increase extracellular PGRN	Intravenous (IV)	Under investigation	[232, 236]
Anti-sortilin antibody	Latozinemab (AL001)	Phase 1	NCT03636204	Block lysosomal delivery of PGRN to increase extracellular PGRN	Intravenous (IV)	Well tolerated, reduced WBC sortilin, and increased plasma and CSF PGRN in healthy volunteers and aFTD-GRN participants.	[232, 236]
Anti-sortilin antibody	AL101	Phase 2	NCT06079190	Block lysosomal delivery of PGRN to increase extracellular PGRN	Intravenous (IV)	Under investigation	No published preclinical data
Anti-sortilin antibody	AL101	Phase 1	NCT04111666	Block lysosomal delivery of PGRN to increase extracellular PGRN	Intravenous (IV) or subcutaneous (SC)	Generally safe and well tolerated, and increased plasma and CSF PGRN in healthy volunteers	No published preclinical data
*GRN* gene therapy	PR006/ LY3884963	Phase 1/2	NCT04408625	AAV-mediated *GRN* gene delivery	Intracisterna magna (ICM)	increased CSF PGRN in all patients	[216]
*GRN* gene therapy	PBFT02	Phase 1/2	NCT04747431	AAV-mediated *GRN* gene delivery	Intracisterna magna (ICM)	Under investigation	[229]
*GRN* gene therapy	AVB-101	Phase 1/2	NCT06064890	AAV-mediated *GRN* gene delivery	Bilateral intrathalamic infusion	Under investigation	No published preclinical data
PGRN protein replacement therapy	DNL593	Phase 1/2	NCT05262023	Brain-penetrant recombinant PGRN biologic	Intravenous (IV)	Under investigation	[210]
Alkalizing agent	Amiodarone	Completed (phase 2)	2011-004571-37	Upregulate *GRN* mRNA	Oral	No significant effects on serum PGRN and disease course	[240]
HDAC inhibitor	FRM-0334	Completed (phase 2a)	NCT02149160	Upregulate *GRN* mRNA	Oral	Safe and well tolerated, but no effects on plasma and CSF PGRN	[244]
Calcium channel blocker	Nimodipine	Completed (Phase 1)	NCT01835665	Increase secreted PGRN	Oral	Safe and well tolerated, but no effects on plasma and CSF PGRN	[238]

Abbreviations: WBC, white blood cell

For FTLD-*GRN*, several other approaches have also been explored to increase PGRN from the intact *GRN* allele and restore its protein levels in the brain. One such approach is targeting sortilin-PGRN interaction to inhibit cellular uptake of PGRN [232–235]. Latozinemab (also known as AL001), an anti-sortilin antibody that blocks sortilin-PGRN interactions and targets sortilin for degradation [232], has demonstrated favorable safety and an increase in plasma and CSF PGRN levels in FTLD-*GRN* participants in a phase 1 clinical trial [236], and is currently in a phase 3 clinical trial (NCT04374136) (Table 3). In addition, AL101, another anti-sortilin antibody, is currently in a phase 2 clinical trial for the treatment of neurodegenerative diseases, including AD and PD (NCT06079190).

Another approach to increase PGRN levels used in a recent study is focusing on microRNAs (miRs) that negatively regulates PGRN protein levels. Antisense oligonucleotides (ASOs) to target the binding site of miR-29b in the 3’ UTR of the human *GRN* mRNA have been shown to effectively increase PGRN translation in human iPSC-derived neurons and in a humanized *GRN* mouse model [237].

Small molecules that can increase PGRN levels have also been explored and some have been tested in clinical trials (Table 3). Nimodipine, an FDA-approved BBB-penetrant calcium channel blocker, increases PGRN levels in *Grn*^+/−^ mice through unknown mechanisms, but did not alter plasma or CSF PGRN levels in a phase 1 clinical trial [238]. V-ATPase inhibitors and alkalizing reagents such as chloroquine and amiodarone have been shown to increase intracellular and secreted PGRN in cell culture via a translational mechanism independent of autophagy, lysosomal degradation, or endocytosis [239]. However, amiodarone administration had no significant effects on serum PGRN and disease course in a pilot phase 2 clinical trial with five FTLD-*GRN* patients [240]. Histone deacetylase (HDAC) inhibitors have been shown to upregulate PGRN transcription in cell culture systems, including iPSC-derived neurons [241–243] but failed to increase plasma and CSF PGRN levels in participants with *GRN* haploinsufficiency in a phase 2a clinical trial [244]. There are preclinical studies reporting that the disaccharide trehalose, BBB-penetrant benzoxazole derivatives, and bromodomain and extra-terminal domain (BET) inhibitors significantly increase PGRN mRNA and protein levels in iPSC-derived neurons [245–247] and in *Grn*^+/−^ mouse brains [245, 246].

As many FTLD-*GRN* cases are caused by nonsense mutations, which result in PTCs in the *GRN* mRNA, another potential treatment strategy may be nonsense suppression therapy, which has been investigated in many other genetic diseases caused by nonsense mutations [248]. Two previous studies have shown that aminoglycosides-induced PTC readthrough upregulates PGRN expression in some but not all nonsense mutations tested in cell culture and preclinical models [249, 250]. It is however known that long-term administration of aminoglycosides causes off-target toxic side effects [248]. Therefore, further investigation will be needed to find safe, effective PTC readthrough agents. Inhibiting nonsense-mediated mRNA decay (NMD) may be an alternative approach. However, a study has reported that ASO-based inhibition of NMD of *Grn*^*R493X*^ mutant mRNA and genetic deletion of the NMD factor *Upf3b* do not increase *Grn* mRNA levels in the *Grn*^*R493X*^ mouse model although the ASOs successfully increases *Grn* mRNA levels in fibroblasts derived from *Grn*^*R493X*^ knockin mice [251].

Beyond FTLD-*GRN* and CLN11, effects of boosting PGRN levels have also been assessed in several mouse models of AD and PD. Lentivirus-mediated PGRN overexpression lowered plaque load and prevents neuronal loss and memory deficits in 5XFAD mice [184]. Intrahippocampal injection of recombinant PGRN protein also reduces hippocampal Aβ deposition in 5XFAD mice [252]. Lentivirus-mediated PGRN overexpression was also effective in reducing plaque burden and synaptic atrophy in Tg2576 APP transgenic mice [253]. For PD, lentivirus-mediated *GRN* gene delivery has been reported to protect against 1-methyl-4-phenyl-1,2,3,6-tetrahydropyridine (MPTP)-induced dopaminergic neuronal loss and locomotor deficits [254]. So far, effects of boosting PGRN levels on tau and α-synuclein pathologies have not been investigated.

## Conclusion and future direction

PGRN boosting therapy is a promising therapeutic strategy for FTLD-*GRN* and CLN11, and also potentially for other neurodegenerative diseases. However, it should be noted that PGRN overexpression has long been known to be associated with many tumors [255, 256]. Safety and adverse effects of long-term treatment therefore will need to be assessed carefully (BOX 1). In terms of the sortilin antibody approach, it is also important to note that a recent study has shown that lysosomal PGRN in neurons and the granulins/PGRN ratio are significantly decreased in sortilin KO mouse brain despite increased total PGRN levels in the brain [105]. Therefore, potential effects of selective loss of neuronal PGRN and granulins in subject treated with therapeutic anti-sortilin antibodies will need to be considered. So far, little is known about the physiological or pathological role(s) of granulins versus PGRN in the brain. A recent preclinical study has shown that AAV-mediated expression of granulin 2/F or 4/A rescues several phenotypes of *Grn*^−/−^ mice [212], revealing a protective role of granulins in the brain. Further studies are necessary to understand the exact functions of individual granulins (BOX 1).

**Box 1:** Key outstanding questions regarding PGRN and TMEM106B in the field

What are functions of individual granulin peptides versus PGRN in the brain?
How do TMEM106B variants affect FTLD-GRN and other neurodegenerative diseases?
Do PGRN and TMEM106B exert their deleterious/protective effects predominantly in neurons versus glia?
What are the mechanisms of amyloid fibril formation of C-terminal fragments of TMEM106B?
Do the TMEM106B fibrils directly contribute to pathophysiology of neurodegenerative diseases?
Can we establish a preclinical model that fully reproduces effects of PGRN haploinsufficiency?
What are the mechanisms by which PGRN deficiency causes alteration in lipids such as BMP and glycosphingolipids?
Is the lipid alteration caused by PGRN or TMEM106B deficiency directly responsible for PGRN- or TMEM106B-associated neurological diseases?
Do PGRN-boosting therapies have side effects?

TMEM106B has not been considered as a therapeutic target because preclinical studies have found that both complete loss and overexpression of TMEM106B cause lysosomal abnormalities in the brain. It is important to note however that, given their presence in multiple neurodegenerative diseases as well as in aged brains, if TMEM106B amyloid fibrils are found to have a pathological role, preventing formation of and/or removing these amyloid fibrils may be a therapeutic strategy for various neurodegenerative diseases. Thus, better understanding of the TMEM106B fibrils through relevant in vitro and in vivo models recapitulating formation of TMEM106B amyloid fibrils will be necessary (BOX 1). Importantly, there is currently no standardized criteria and methods for identification of the TMEM106B fibrils. While determining cryo-EM structure proves the presence of the TMEM106B fibrils, immunohistochemistry studies using antibodies against luminal region of TMEM106B could potentially detect abnormally overexpressed full-length TMEM106B or cleaved but non-fibrillar luminal region of TMEM106B. In addition, the molecular weight of the sarkosyl-insoluble CTF in immunoblot analysis varies between published studies [83, 156–158, 170]. More specific antibodies, probes and/or image analyses to detect the TMEM106B fibrils may help solving these issues.

The recent discovery of an involvement of PGRN and TMEM106B in lipid metabolism by directly regulating several lipid classes such as GlcCer, BMP, gangliosides, and GalCer is a significant advance. It is interesting to note that both PGRN and TMEM106B appear to be involved in glycosphingolipid metabolism (Fig. 3). PGRN regulation of GlcCer has been demonstrated to be critical for developing Gaucher disease [57] and potentially for synucleinopathy and tauopathy [49]. However, it remains unclear whether other lipids contribute to brain aging or the pathophysiology of PGRN- or TMEM106B-associated neurological diseases (BOX 1). In addition, lipid-droplet-accumulating *Grn*^−/−^ microglia were recently shown to be defective in phagocytosis [222]. In contrast, several previous studies have reported that PGRN deficiency enhances phagocytosis and/or synaptic pruning in macrophages and microglia [16, 52, 257–259]. Resolving these issues and apparent contradictions may lead to development of novel alternative therapeutic approaches targeting common lipid pathways shared by various neurodegenerative diseases.

Finally, it is important to note that although commonly used *Grn*^−/−^ and *Tmem106b*^−/−^ mice and their crosses have been valuable tools to understand physiological functions of PGRN and TMEM106B and their general roles under pathological conditions, these mice with complete loss of PGRN or TMEM106B do not accurately model human diseases associated with PGRN and TMEM106B except for CLN11. Thus, to better understand disease mechanisms and to facilitate the development of novel therapies, it is necessary to establish more relevant preclinical models recapitulating effects of human *GRN* mutations or variants and the *TMEM106B* risk haplotype (BOX 1). Importantly, it remains unclear how the *TMEM106B* haplotype modifies a disease risk across various neurodegenerative diseases as well as brain aging (BOX 1). The *TMEM106B* risk haplotype has been suggested to increase TMEM106B protein levels [7, 61, 68, 79, 83]. However, this needs to be further verified experimentally by determining the functional variant(s) on the haplotype and elucidating the underlying mechanism. The verification is especially important because (1) inconsistent results have been obtained in its effect on mRNA levels [7, 63, 68, 79–81] and in the protein stability of the T185S protective variant [61, 135, 150, 152, 153], (2) cell culture and mouse studies have shown that both loss and overexpression of TMEM106B negatively affect some aspects of lysosomal function and exacerbate phenotypes of PGRN-deficient mice [89, 92, 93, 134–137, 143, 191, 195], and (3) a few studies have reported potential functional impacts of the T185S protective variant [150, 152]. It also remains ill-defined whether PGRN and TMEM106B exert their deleterious/protective effects predominantly in neurons versus glia and whether PGRN and TMEM106B interact intracellularly or intercellularly between different cell types to affect neurodegeneration and brain aging (BOX 1). For example, PGRN-deficient microglia may specifically influence neurons with increased TMEM106B levels, similar to the results shown in a previous study [178]. Further studies using co-culture, 3D culture, or xenotransplantation of human iPSC-derived neurons and glia generated from patients with or without *GRN* mutations and/or with the *TMEM106B* risk or protective haplotype may achieve the goal. Once the link between the risk haplotype and increased TMEM106B protein levels is confirmed, animal models with increased TMEM106B levels like the recent human TMEM106B transgenic mice [199] will be also useful.

## Data Availability

No datasets were generated or analysed during the current study.

## Abbreviations

AAV: Adeno-associated virus
Aβ: Amyloid-β
AD: Alzheimer’s disease
ADNI: Alzheimer’s Disease Neuroimaging Initiative
AEP: Asparagine endopeptidase
ALS: Amyotrophic lateral sclerosis
AP1: Accessary protein 1
ASO: Antisense oligonucleotide
BBB: Blood-brain barrier
BMP: Bis(monoacylglycero)phosphate
CBD: Corticobasal degeneration
CHMP2B: Charged multivesicular body protein 2B
CLN11: Neuronal ceroid lipofuscinosis type 11
CNS: Central nervous system
Co-IP: Co-immunoprecipitation
Cryo-EM: Cryo-electron microscopy
CSF: Cerebrospinal fluid
CTCF: CCCTC-binding factor
CTF: C-terminal fragment
DKO: Double knockout
ESCRT-III: Endosomal sorting complex required for transport-III
FTLD: Frontotemporal lobar degeneration
FTLD-TDP: FTLD with TDP-43 inclusions
FTLD-*GRN*: FTLD with *GRN* mutations
GALC: Galactosylceramidase
GalCer: Galactosylceramide
GCase: β-Glucocerebrosidase
GlcCer: Glucosylceramide
GlcSph: Glucosylsphingosine
GWAS: Genome-wide association study
HDAC: Histone deacetylase
HexA: β-Hexosaminidase A
HLD: Hypomyelinating leukodystrophy
IP: Immunoprecipitation
IP-MS: Immunoprecipitation-mass spectrometry
iPSC: Induced pluripotent stem cell
KO: Knockout
LATE-NC: Limbic-predominant age-related TDP-43 encephalopathy neuropathological change
LBD: Lewy body dementia
LC-MS/MS: Liquid chromatography tandem mass spectrometry
LD: Linkage disequilibrium
LEA-2: Late embryogenesis abundant-2
LRP1: Low-density lipoprotein receptor-related protein 1
MAP6: Microtubule-associated protein 6
miR: MicroRNA
MPTP: 1-Methyl-4-phenyl-1,2,3,6-tetrahydropyridine
M6PR: Mannose 6-phosphate receptor
NMD: Nonsense-mediated mRNA decay
OCD: Obsessive compulsive disorder
PD: Parkinson’s disease
PGRN: Progranulin
PSAP: Prosaposin
PSP: Progressive supranuclear palsy
PTC: Premature termination codon
RGC: Retinal ganglion cell
SNP: Single-nucleotide polymorphism
SPR: Surface plasmon resonance
TAGs: Triacylglycerols
TDP-43: TAR DNA-binding protein 43
TFEB: Transcriptional factor EB
TfR: Transferrin receptor
V-ATPase: Vacuolar-ATPase
VPS10: Vacuolar protein sorting 10
WBC: White blood cell
Y2H: Yeast two-hybrid
